# Nutrition Label Literacy, Socio-Demographic Correlates, and Perceived Barriers Among Adult Consumers: A Cross-Sectional Study from Islamabad, Pakistan

**DOI:** 10.3390/nu18213689

**Published:** 2026-11-07

**Authors:** Saeed Ahmad Noor, Humaira Mahmood, Abdul Momin Rizwan Ahmad, Jamshaid Ibrahim, Saira Zafar, Syed Hassan Bin Usman Shah, Juweria Abid, Yasir Waheed, Saleh A. Alsanie

**Affiliations:** 1 School of Public Health, Health Services Academy, Islamabad 44000, Pakistan; saeedahmad.nut@gmail.com (S.A.N.); humaira.talha.7@gmail.com (H.M.); drsairaasifiqbal@gmail.com (S.Z.); 2 Department of Human Nutrition and Dietetics, NUST School of Health Sciences, National University of Sciences & Technology (NUST), Sector H-12, Islamabad44000, Pakistan; juweria.abid@nshs.nust.edu.pk; 3 Department of Health Sciences, University of York, York YO10 5DD, UK; 4 Institute of Food Science and Technology, Khwaja Fareed University of Engineering & Information Technology, Rahim yar Khan 64200, Pakistan; jamshaidibrahim129@gmail.com; 5 Department of Public Health, PRIME Consulting, Islamabad 44000, Pakistan; hassanbinusman@primeconsulting.com.pk; 6 NUST School of Health Sciences, National University of Sciences and Technology (NUST), Sector H-12, Islamabad 44000, Pakistan; yasir.waheed@nshs.nust.edu.pk; 7 Irfan Suat Gunsel Operational Research Institute, Near East University, 99138 Nicosia, Turkey; 8 Advanced Research Centre, European University of Lefke, 99790 Lefke, Northern Cyprus, Turkey; 9 Department of Health Sciences, VIZJA University, 01-043 Warsaw, Poland; 10 Department of Basic Health Sciences, College of Applied Medical Sciences, Qassim University, Buraydah 52581, Saudi Arabia; 11 Department of Clinical Nutrition, Medical City, Qassim University, Buraydah 52581, Saudi Arabia

**Keywords:** nutrition label literacy, food label literacy, front-of-package labeling, health literacy, socio-demographic factors, Pakistan, cross-sectional study

## Abstract

**Background/Objectives:** Pakistan has not adopted front-of-package warning labels, and no validated, task-based instrument had previously measured nutrition label literacy among Pakistani adults. This study aimed to measure nutrition label literacy among adult grocery consumers in Islamabad, describe performance on individual tasks, identify socio-demographic correlates, and examine whether self-reported barriers differ by measured literacy. **Methods:** In this cross-sectional study, we recruited 400 adult consumers (73.0% response rate) at six retail sites spanning three socioeconomic tiers in Islamabad Capital Territory, Pakistan, using systematic intercept sampling. Label literacy was measured with a culturally adapted, bilingual Urdu/English version of the Newest Vital Sign (NVS-PK). Socio-demographic correlates were examined with multiple linear and binary logistic regression, and self-reported barriers were ranked and compared across literacy categories. **Results:** Mean NVS-PK score was 1.89 of 6 (SD = 1.71); 81.0% of participants scored in the limited-or-marginal range and only 19.0% reached adequate literacy. Performance was weakest on arithmetic tasks (14.8–29.2% correct) and strongest on a document-based allergen-identification task (66.8% correct). Educational attainment was the strongest independent correlate: compared with Intermediate education or below, postgraduate participants scored significantly higher (B = 1.64, 95% CI 1.17–2.10, *p* < 0.001) and had markedly higher odds of adequate literacy (AOR = 8.72, 95% CI 3.88–19.60, *p* < 0.001). Technical terminology (70.2%) and time pressure (64.0%) were the most reported barriers to label use. **Conclusions:** Most adults sampled in Islamabad could not adequately use a standard nutrition label, and the deficit was primarily arithmetic rather than reading.

## 1. Introduction

Health literacy is now recognized as a social determinant of health, not simply a byproduct of general schooling [1]. It is the ability to find, understand and use health information, and it is not a fixed personal trait: it can change, shaped by education, social position, and how much the health system and the food market demand of a person [1]. Its most basic level is functional literacy, the reading and number skills needed to understand information; interactive and critical literacy build on this, allowing people to apply information and judge it [1]. A consumer who cannot work out how much sodium a product adds to the daily limit is in no position to judge the health claims printed beside that number [2].

Nutrition literacy is the part of health literacy that deals with food, diet and nutrients, and it has the same functional, interactive and critical levels [3]. Nutrition label literacy, the skill measured in this study, is narrower still: the ability to find, read, calculate and interpret the information printed on a packaged food label [2,4]. Because it depends mostly on functional skills, it is where gaps in reading and number skills show up when people shop for food.

Over the last forty years, diets around the world have moved away from simple, home-prepared foods towards factory-made, ultra-processed products [5]. A review of many meta-analyses links high intake of these foods to death from any cause, heart disease, type 2 diabetes and obesity [6]. The change has been fastest in low- and middle-income countries. In these countries, packaged foods reached ordinary households before the rules and labelling systems meant to help people judge them [7]. Processed food products do not show what they contain, so the label is the main source of that information. When labels are hard to read and understand, consumers fall back on the packaging to judge how processed a product is [8].

For this reason, nutrition labelling at the point of sale has been adopted worldwide as a low-cost way to help people choose better food [7]. In recent years, many countries adopted front-of-package (FOP) warning, because these are easier to read than a full nutrition panel and have been shown to change what people buy [9,10]. Where no such scheme exists, the back-of-pack nutrition information panel remains consumers’ main source of label information. Using it correctly is a multi-step task. A consumer must locate the serving size, recognize that one serving is usually smaller than the whole, multiply the nutrient values by the number of servings, and compare percentage daily values against a standard diet [11]. Research shows that it is these calculations, rather than the reading itself, where people fail. In a national survey in the United States, about 42% of adults could not work out the calories saved by skipping one serving, and 41% could not calculate a percentage daily value [4]. A clinic-based study found that patients with weaker number skills did worse only on the label questions that required calculation, not on those that required simple reading [2].

These calculation problems are not limited to the United States. Wherever label comprehension has been tested rather than self-reported, results have been poor even in high-income countries [12,13]. Across five countries, people reported understanding labels better than they did, and the gap was widest among those with lower income and less education [14]. A review of ten African countries found education and income to be the factors most often linked to label understanding, and technical wording to be a leading barrier [15]. Evidence from South Asia is limited and rests mainly on self-report: a scoping review from India [16], a study in which higher-income Indian women understood nutrition information better than men [17], and a knowledge–attitude–practice survey of consumers in South Chennai [18]. In Pakistan, one survey found that only 13.1% of consumers always read the nutrition facts printed on food labels [19].

Pakistan has one of the heaviest burdens of diet-related disease in the world, so whether its consumers can use nutrition information is a practical public health question. The International Diabetes Federation puts adult diabetes prevalence at 26.7%, among the highest reported anywhere [20]. Population studies suggest that about 34% of urban adults have high blood pressure [21], and non-communicable diseases cause most deaths in the country [22]. Food labels in Pakistan may be in English or Urdu, and since 2019 imported packaged foods must show nutritional values in both languages [23]. Locally made products have no such requirement, and their nutrition panels are printed almost entirely in English, although Urdu is the national language and the one most people use every day. Pakistan has also not adopted a front-of-package scheme [24], so the back-of-pack panel remains the main source of nutrition information. Both national [24] and regional [25] reviews have described this as a policy gap.

Islamabad differs from the rest of the country. Literacy among people aged 10 years and over is 84.0%, compared with 60.7% nationally, and 46.9% of its population is urban, against 38.8% [26]. This makes the capital a conservative setting for this study: because most consumers can read, low scores are more likely to reflect difficulty with the calculations and the label itself than an inability to read.

Most regional and Pakistani studies rely on self-reported data. They use knowledge–attitude–practice surveys, which ask people whether they read and understand labels rather than testing whether they can. Answers to such questions are affected by social desirability bias, and multi-country data show that people report understanding more than they can demonstrate [14]. Task-based tools avoid this problem by asking people to perform. The Newest Vital Signs NVS, developed by Weiss and colleagues, gives the respondent a real food label and six standard questions, and is widely used as a quick measure of functional health literacy [27]. Its label comes from a one-pint ice cream pack, a product that Pakistani households rarely buy and do not find familiar. It requires proper cross-cultural adaptation [28], and this has been done once before for Urdu speakers in Pakistan [29].

A recent study measured general nutritional literacy among adults living in two marginalized settlements in Islamabad. Using a purpose-built 14-item self-report tool, it found that 89.1% had poor nutritional literacy, and education was the factor most strongly associated with the outcome [30]. Those authors noted that task-based tools developed in high-income countries may not suit Pakistani marginalized communities. To the best of our knowledge, no study has yet used a validated, task-based tool to measure nutrition label literacy, as distinct from general nutrition knowledge, among ordinary adult consumers in Pakistan, so it remains unknown which specific label-reading tasks are hardest for this population, and whether education remains the dominant correlate once measured, task-based performance replaces self-report. It is also unknown whether the barriers consumers report match their measured performance on label tasks. Without such evidence, regulators cannot tell whether the problem lies with the consumer’s skills or with the design of the label, and so cannot choose sensibly between public education, label redesign and a change in the law.

This study aimed to measure how well adult grocery consumers in Islamabad can read, understand and use the information on a standard nutrition label, using a culturally adapted NVS at three types of retail outlet. Its specific objectives were to describe how well participants answered each of the six tasks, to examine which socio-demographic characteristics were associated with performance, and to rank the barriers consumers reported and test whether these differed by measured literacy.

## 2. Materials and Methods

### 2.1. Design and Settings

This was a cross-sectional study of adult grocery consumers in the Islamabad Capital Territory (ICT), Pakistan, carried out at six retail sites from July 2026 to August 2026. We grouped the sites into three tiers based on the location and type of each market. Islamabad’s sectors have been divided by income since the city was planned, with larger houses and plots in the F-sectors [31]. Official property valuation rates follow the same order [32]. Tier 1 covered two high-income grocers, Kohsar Market (F-6) and Centaurus Mall (F-8), located in two of the city’s highest-valued sectors (*n =* 134). Tier 2 covered two mixed-income sites *(n* = 133): I-8 Markaz, which serves a middle-income sector, and Metro Cash & Carry, a wholesale store whose customers are mostly middle-income households and small shopkeepers from across the city. Tier 3 covered two lower-middle-income markets (*n* = 133): Bhara Kahu, an unplanned peri-urban area outside the CDA sectors that is valued as rural land [32], and G-9 Markaz (Karachi Company), a low-cost market. We report the study following the STROBE guidelines for observational research [33,34].

### 2.2. Participants and Sampling

Eligible participants were adults aged 18–65 living, working or studying in the ICT who bought packaged food independently at least weekly. We excluded people with formal nutrition, dietetics, medical or allied-health training (to avoid inflated scores), severe uncorrected vision or cognitive impairment, inability to read Urdu or English, and on-duty retail staff.

No sampling frame exists for Islamabad consumers, so we used systematic intercept sampling: enumerators approached every 5th adult crossing a fixed point near each store’s exit, with equal quotas per retail tier. Refusals were logged and the next person approached, then the 1-in-5 count resumed [35]. A 1:1 gender quota corrected for intercept sampling’s typical under-recruitment of women in South Asia. Of 548 approached, 400 consented and completed the assessment (73.0% response rate; 55.0% male, 45.0% female); the remaining 148 either declined or stopped before finishing, and both were counted as non-respondents.

Minimum sample size, by Cochran’s formula for a single proportion (Z = 1.96, *p* = 0.5, e = 0.05), was *n =* 384 [36], rounded to *N =* 400 and split evenly across tiers (133–134 each) for adequate power in the planned regressions and ANOVAs (>85% power for f = 0.18, d = 0.35 at α = 0.05) [37].

The interviewer explained the study and obtained written informed consent by signature or thumbprint. Participants were told that taking part was voluntary and that they could stop without giving a reason. The questionnaire was administered face to face, mostly in Urdu, and answers were recorded on paper forms; each interview took about 8–12 min. It was anonymous: each form carried only a participant code, and no names, phone numbers or other identifiers were recorded. Forms were checked for completeness before the participant left, so the 400 completed assessments had no missing data.

### 2.3. Instrument

We measured label literacy with a bilingual Urdu/English adaptation of the Newest Vital Sign (NVS-PK), developed for this study. The original NVS scores six tasks against an ice-cream-container label [27]; we substituted a 150 g biscuit pack. Task structure, comprising one multiplication, one division, one subtraction, one percentage-daily-value calculation, and two allergen-identification tasks, was kept identical to preserve comparable difficulty.

As a full adaptation rather than a translation, it followed a six-stage process: forward translation, synthesis, blind back-translation, Delphi review at the Health Services Academy, a 42-participant pilot, and final sign-off, following established cross-cultural adaptation guidelines [28] and prior Urdu NVS adaptations in Pakistan [29].

The instrument had three parts: Part A, sociodemographic/shopping data and a label-reading confidence item; Part B, the six-item NVS-PK battery; and Part C, a 10-item barrier checklist on which participants could mark any number of barriers. Each NVS-PK item was scored 1 if correct and 0 if not, giving a total score from 0 to 6, interpreted with the original cut-offs: 0–1 high likelihood of limited literacy, 2–3 possible limited literacy, and 4–6 adequate literacy [27]. Items B1–B4 formed an applied-numeracy subscale (0–4) and B5–B6 a document-literacy subscale (0–2). The original NVS showed good internal consistency (Cronbach’s α = 0.76) [27], and α was 0.733 in the present sample. Socioeconomic position was scored with the Modified Kuppuswamy scale (household head’s education, occupation, and monthly income) [38].

### 2.4. Statistical Analysis

Internal consistency was assessed with Cronbach’s alpha. Prevalence estimates and item-level proportions are reported with exact (Clopper–Pearson) binomial 95% confidence intervals [39]. Unadjusted NVS-PK scores were compared across groups using independent-samples t-tests and one-way ANOVA; because Levene’s test showed unequal variances for education, income, socioeconomic class and retail tier, we report Welch’s F with Games-Howell pairwise comparisons for these variables, and the Jonckheere–Terpstra test for ordered trends.

Independent correlates of literacy were identified with two-block multiple linear regression (age, gender, education, income; then retail tier) on total NVS-PK score, and binary logistic regression on adequate versus non-adequate literacy (adjusted odds ratios, Hosmer–Lemeshow test, Nagelkerke R^2^). A random-intercept model (retail site as grouping factor) estimated site-clustering effects. Robustness to any single site was checked by refitting the linear model six times, leaving out one site each time. Agreement between self-rated confidence (three levels) and NVS-PK category was assessed with Cohen’s kappa. Barrier-checklist items were compared with Cochran’s Q (equality of reporting rates) and chi-square/Cramér’s V (variation by literacy category).

All tests were two-sided (α = 0.05), using IBM SPSS Statistics v26 (IBM Corp., Armonk, NY, USA).

## 3. Results

### 3.1. Sample and Nutrition Label Literacy Levels

Out of 548 consumers approached, 400 completed the assessment, a 73.0% response rate (Section 2.2). Participants ranged from 18 to 64 years (M = 33.55, SD = 8.12); 55.0% were male. Table 1 summarizes the sample.

**Figure 1:**
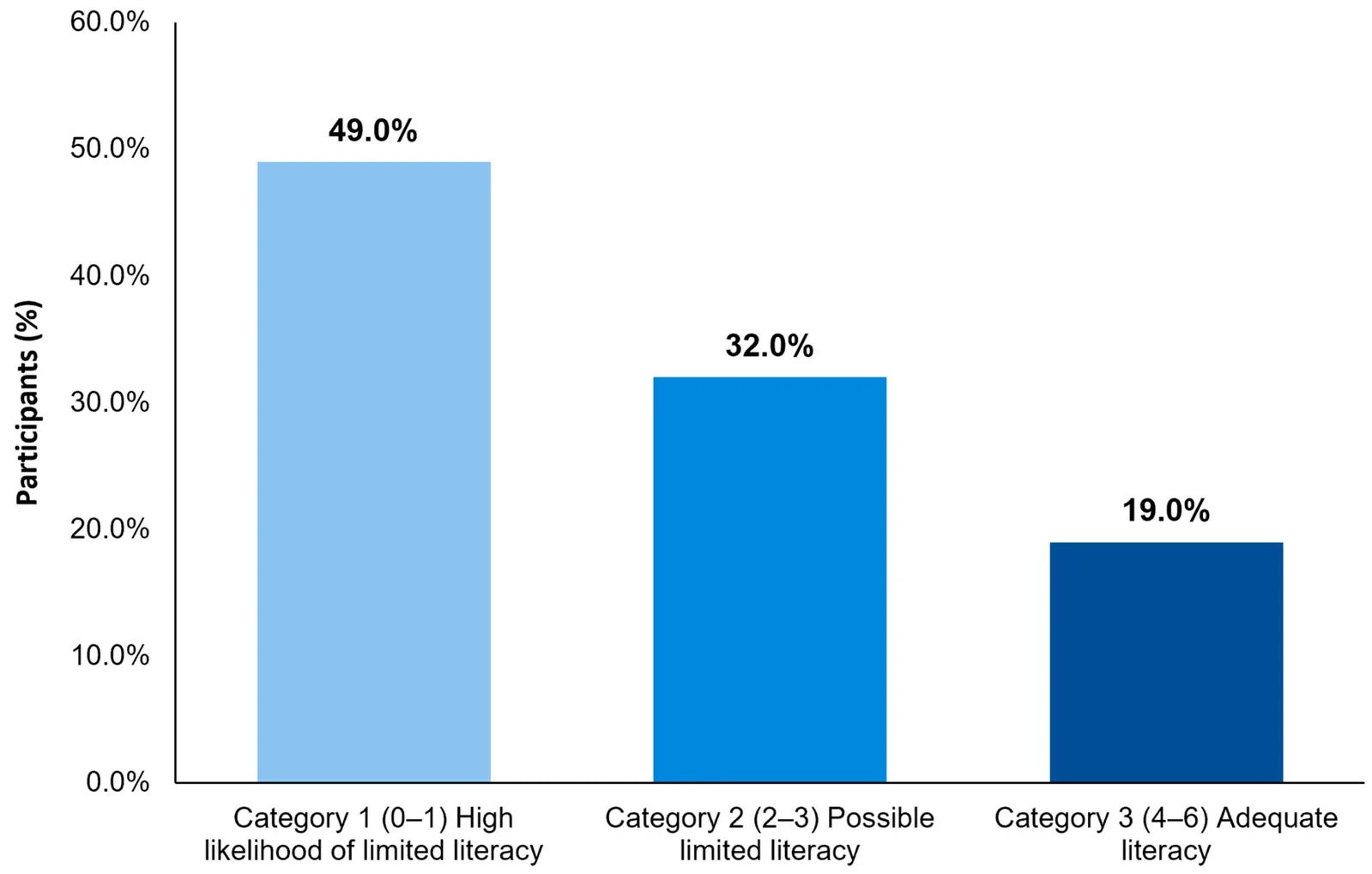
Distribution of participants across the three validated NVS-PK literacy categories (*N =* 400). Bars show the percentage in each category; darker shading indicates higher literacy.

Mean NVS-PK score was 1.89 (SD = 1.71) of 6. Applying the validated cut-offs, 81.0% (*n =* 324; 95% CI 76.8–84.7) showed limited or marginal literacy: 49.0% (n = 196) scored in the highest-risk category (0–1) and 32.0% (n = 128) in the Intermediate category (2–3). Only 19.0% (*n =* 76; 95% CI 15.3–23.2) reached adequate literacy (4–6) (Table 2; Figure 1). Internal consistency was acceptable, Cronbach’s α = 0.733; scores were lower on the four-item applied-numeracy subscale (0.92 of 4) than the two-item document-literacy subscale (0.97 of 2).

Item difficulty varied sharply, Cochran’s Q(5) = 406.96, *p* < 0.001: allergen identification was easiest (66.8% correct) and expressing sodium as a percentage of the daily limit was hardest (14.8% correct). Self-rated confidence correlated only weakly with measured performance (Spearman ρ = −0.304, *p* < 0.001); of the 53.5% who rated themselves confident, 73.4% nonetheless scored in the limited-or-marginal range.

### 3.2. Socio-Demographic Associations (Unadjusted)

Bivariate analysis showed that educational attainment, household income, socioeconomic class and retail tier were each significantly associated with NVS-PK score (all *p* < 0.001; Table 1); gender (*p* = 0.780) and marital status (*p* = 0.916) were not, and age showed only a weak inverse association (r = −0.106, *p* = 0.034). Mean scores rose monotonically with education, from 1.18 (Intermediate or below) to 2.60 (Bachelor’s) to 3.28 (postgraduate). Tier 1 consumers scored highest (M = 2.61) and Tier 3 consumers lowest (M = 1.17).

### 3.3. Multivariable Analysis

In the fully adjusted linear model (Table 3, Model 2), compared with Intermediate education or below, participants with a Bachelor’s degree (B = 1.06; 95% CI 0.69, 1.43; *p* < 0.001) and postgraduate education (B = 1.64; 95% CI 1.17, 2.10; *p* < 0.001) scored significantly higher on the NVS-PK. Compared with household income below PKR 50,000, the highest income band was independently associated with a higher score (B = 0.41; 95% CI 0.01, 0.81; *p* = 0.043). Compared with Tier 1, Tier 3 consumers scored significantly lower (B = −0.59; 95% CI −0.99, −0.19; *p* = 0.004). Age and gender were not significantly associated with score after adjustment. The model explained 27.1% of the variance (adjusted R^2^ = 0.271).

In the fully adjusted logistic model (Table 4), compared with Intermediate education or below, a Bachelor’s degree (AOR = 4.23; 95% CI 2.04, 8.79; *p* < 0.001) and postgraduate education (AOR = 8.72; 95% CI 3.88, 19.60; *p* < 0.001) were associated with significantly higher odds of adequate literacy. Compared with Tier 1, Tier 2 consumers had significantly lower odds (AOR = 0.43; 95% CI 0.22, 0.84; *p* = 0.013); the Tier 3 contrast was not significant (AOR = 0.58; 95% CI 0.26, 1.32; *p* = 0.196). The model fitted the data well (Hosmer–Lemeshow *p* = 0.486; Nagelkerke R^2^ = 0.253).

A random-intercept model showed modest clustering by retail site (ICC = 0.143); adjusting for socio-demographic covariates reduced the between-site variance by about 87%, indicating that most of this clustering reflected consumers’ socioeconomic composition rather than the retail environment itself.

### 3.4. Perceived Barriers to Label Use

**Figure 2:**
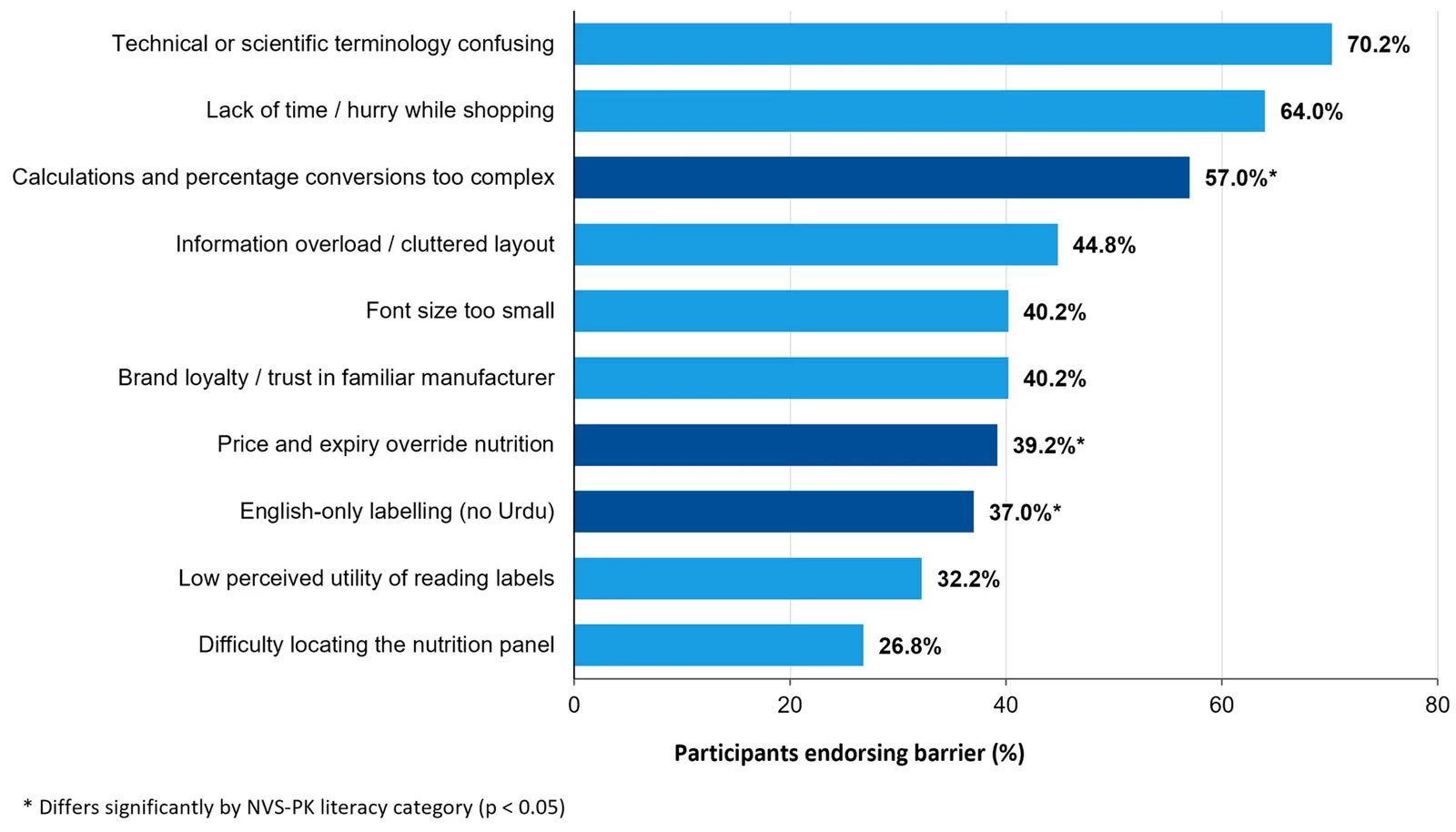
Ranked prevalence of the ten perceived barriers to nutrition label use (*N =* 400). Darker bars mark the three barriers whose endorsement rate differed significantly by NVS-PK literacy category (*p* < 0.05).

Participants endorsed a mean of 4.52 barriers (SD = 1.51) from the 10-item checklist; endorsement rates differed sharply across items, Cochran’s Q(9) = 284.73, *p* < 0.001. Technical terminology was the most common barrier (70.2%), followed by time pressure while shopping (64.0%) and calculation complexity (57.0%) (Table 5; Figure 2). Only three barriers varied significantly by literacy category: calculation complexity (72.4% among the lowest-literacy category versus 25.0% among those with adequate literacy, χ^2^(2) = 51.97, *p* < 0.001), English-only labelling (49.0% vs 19.7%, *p* < 0.001), and price priority (43.9% vs. 22.4%, *p* = 0.004). The two most prevalent barriers, terminology and time pressure, did not differ by literacy category, suggesting these reflect shopping conditions common to all consumers rather than deficits specific to lower-literacy participants.

## 4. Discussion

Most adults recruited at these six Islamabad retail sites could not complete most tasks on a standard nutrition information panel: 81.0% scored in the limited-or-marginal range, and even among postgraduate-educated participants the mean score (3.28) fell short of the threshold for adequate literacy. This is high compared with studies using the same six tasks and the same cut-offs: 54% in the limited-or-marginal range at a United States family medicine clinic (mean 3.9) [12], 42.1% with the Turkish-validated NVS (mean 3.76) [13], and 58% below the adequate threshold with a linguistically validated Croatian version (mean 3.34) [40]. The mean of 1.89 here is more than a full point below the lowest of the three.

The closest comparison in design is Paiva et al. [41], who applied a cross-culturally adapted NVS to 1,544 residents of Portugal and found limited health literacy in 72.9%. It recruited from the general population rather than a clinic, and its figure sits much nearer the 81.0% found here. The two community-based estimates are the two highest of the five, so part of the difference between the present result and the earlier NVS literature may reflect who was approached rather than where.

Studies from the Eastern Mediterranean and South Asia that tested comprehension rather than asking about it report the same picture, though none used the NVS and none is directly comparable. Among 939 Jordanian adults, only 26.4% were comprehensive label users, and mean performance on four comprehension questions was 1.39 of 4, despite favourable attitudes and reported habits [42]. Among 1238 Bangladeshi consumers, more than half had limited practical understanding of the nutrient information on a label, while 91.9% rated labels as important [43]. In Pakistan, Ahmad et al. [30] reported poor nutritional literacy in 89.1% of adults in two marginalized Islamabad settlements, a more disadvantaged population, assessed with a self-developed instrument. What runs through all four sets of findings is the difference between what consumers say about labels and what they can do with them.

The deficit was greatest in numeracy not reading. The allergen-identification item, a document-scanning task, was answered correctly by 66.8% of participants; the sodium-percentage item by only 14.8%. The allergen advantage was partly a matter of format: B5 needed only a yes or no answer, and 147 of the 267 participants who answered it correctly could not then name the ingredient (B6, 30.0% correct). Calculation items were also the hardest in nationally representative US data, where large minorities of adults could not calculate a daily-value percentage or the calorie impact of a serving [4], and in clinic-based evidence that lower-numeracy patients do worse specifically on calculation-based tasks [2]. This suggests the panel’s numeracy demands drive the difficulty, not a Pakistan-specific deficit, though this design cannot test that directly.

The same difference appeared within individual participants (κ = 0.129): more than half rated themselves confident, yet most of those individuals scored in the limited-or-marginal range. Comparable gaps have been reported between self-reported and functional label understanding across five countries [14], and between physicians’ beliefs about patient comprehension and their patients’ actual scores [12]. Communication strategies that assume consumers recognize their own comprehension gaps are unlikely to reach the majority who do not.

Educational attainment was the strongest independent correlate of literacy: compared with Intermediate education or below, a bachelor’s degree and postgraduate education were each associated with a higher score. Education carried the same weight in the Portuguese [41], Croatian [40], Bangladeshi [43] and Pakistani [30] samples, and international reviews name it and income as the characteristics most consistently linked to label understanding [15]. Jordan is the exception: education there was only borderline and being female and being the household’s grocery buyer were stronger predictors [42]. Gender was not associated with score in the present sample. Men and women in the sample had similar levels of education, which may explain why no gender gap appeared. Household income kept a modest independent association at its highest band, and retail tier remained associated with score, though socio-demographic covariates reduced the between-site variance by about 87%, so most of the clustering reflected the socioeconomic mix at each site rather than the retail environment itself. In the logistic model the Tier 3 contrast was not significant, but only 13 Tier 3 participants reached adequate literacy, so this estimate was imprecise.

The barrier analysis adds a finding the prevalence ranking alone would miss. Technical terminology and time pressure were the two most cited barriers, consistent with international reports naming terminology and complexity as leading obstacles [8,15], yet both were reported at statistically indistinguishable rates by participants who could and could not complete the numeracy tasks. That the two most common obstacles were not tied to individual numeracy is consistent with the argument that relying on back-of-package panels as the sole regulatory mechanism disadvantages consumers regardless of individual capability [10].

This study did not test front-of-package labelling and cannot show that a different format would perform better here. Such schemes have reduced the energy, sodium and sugar content of household purchases where implemented [44,45], and the comparative evidence now extends to the region: among 2509 Saudi consumers shown five formats, the interpretive Nutri-Score best improved correct ranking of products by nutritional quality (OR = 5.81) and produced healthier choices than the numeric Guideline Daily Amount format (OR = 1.96, *p* = 0.003) [46]. Regional reviews already recommend front-of-package implementation across the Eastern Mediterranean [25]. A format that does the calculation for the consumer addresses the specific failure measured here.

English-only labelling was reported as a barrier by 37.0% of participants overall but by 49.0% of those with the lowest literacy, and was patterned significantly by education and income, despite Islamabad’s comparatively high English literacy, which likely means the national picture understates it. These findings support considering an interpretive front-of-package label for Pakistan, preferably bilingual and tested with local consumers.

Strengths of this study include a validated, task-based instrument adapted into Urdu, systematic intercept sampling with a gender quota across three socioeconomic retail tiers, complete data for all 400 participants, and leave-one-site-out sensitivity checks. There were, however, several limitations. The cross-sectional design precludes causal inference. Participants came from six selected retail sites, so the 81.0% figure is not a national estimate; recruitment at modern outlets and Islamabad’s higher literacy both mean limited literacy is likely higher elsewhere in Pakistan. Comparisons with other NVS studies are descriptive, as they differ in sampling frame, setting and stimulus product. Non-response linked to time pressure cannot be excluded, and clustering within six sites (ICC = 0.143) limits precision. Barriers and confidence were self-reported to enumerators and may reflect social desirability, income was self-reported, and the checklist did not cover interest in healthy eating, cultural food habits or digital food information. Future studies should apply the NVS-PK in other cities and rural areas, compare label formats with Pakistani consumers, and test whether easier labels change purchases.

## 5. Conclusions

Fewer than one in five adults recruited at these Islamabad retail sites showed adequate nutrition label literacy, and the deficit centred on arithmetic rather than reading. Education was the strongest correlate of performance but was not enough alone: even postgraduate participants averaged below the adequate-literacy threshold. The two most common barriers, technical terminology and time pressure, were reported equally by participants who could and could not do the underlying calculations, pointing toward changing the label rather than only educating the consumer. These data, together with international evidence, support considering bilingual Urdu/English labelling and an interpretive front-of-package label tested with Pakistani consumers. These findings describe consumers at selected Islamabad retail sites and should not be read as national estimates.

## Figures and Tables

**Table 1:** Characteristics of the sample and association with nutrition label literacy (*N =* 400).

Characteristic	n (%)	NVS-PK Score, Mean (SD)	Test Statistic	*p*
Gender (independent-samples t-test)				
Male	220 (55.0)	1.91 (1.66)	t(398) = 0.28	0.780
Female	180 (45.0)	1.86 (1.77)		
Age group, years (one-way ANOVA)				
18–29	135 (33.8)	2.13 (1.72)	F(2, 397) = 3.14	**0.044**
30–39	171 (42.8)	1.87 (1.69)		
40–65	94 (23.5)	1.56 (1.66)		
Marital status (one-way ANOVA)				
Single/never married	138 (34.5)	1.87 (1.60)	F(2, 397) = 0.09	0.916
Married	256 (64.0)	1.89 (1.75)		
Divorced/widowed/separated	6 (1.5)	2.17 (2.40)		
Educational attainment (Welch ANOVA)				
Intermediate or below	230 (57.5)	1.18 (1.28)	Welch F(2, 133.79) = 60.23	**<0.001**
Bachelor’s degree	110 (27.5)	2.60 (1.75)		
Postgraduate	60 (15.0)	3.28 (1.66)		
Monthly household income (Welch ANOVA)				
Below PKR 50,000	128 (32.0)	1.18 (1.38)	Welch F(2, 239.22) = 29.23	**<0.001**
PKR 50,000–74,999	96 (24.0)	1.61 (1.52)		
PKR 75,000 or above	176 (44.0)	2.55 (1.77)		
Socioeconomic class, Modified Kuppuswamy (Welch ANOVA)				
Lower/upper-lower	104 (26.0)	0.95 (1.26)	Welch F(3, 119.90) = 35.17	**<0.001**
Lower-middle	147 (36.8)	1.57 (1.46)		
Upper-middle	118 (29.5)	2.75 (1.72)		
Upper	31 (7.8)	3.26 (1.73)		
Retail tier (Welch ANOVA)				
Tier 1 (high-income)	134 (33.5)	2.61 (1.79)	Welch F(2, 262.84) = 26.43	**<0.001**
Tier 2 (mixed-income)	133 (33.3)	1.87 (1.56)		
Tier 3 (lower-middle-income)	133 (33.3)	1.17 (1.44)		

NVS-PK, culturally adapted Newest Vital Sign—Pakistan (range 0–6). Welch’s F is reported for education, household income, socioeconomic class and retail tier because Levene’s test indicated unequal variances; Fisher’s F gave the same conclusions in every case. Age was also examined as a continuous variable (Pearson r = −0.106, *p* = 0.034). Bold *p*-values indicate *p* < 0.05. All associations shown are unadjusted; characteristics remaining significant after mutual adjustment are shown in Section 3.3.

**Table 2:** NVS-PK score distribution and item-level performance (*N =* 400).

Item	Skill or Concept Evaluated	Correct/In Category, n (%)	95% CI
Literacy category (validated cut-offs)			
Category 1: high likelihood of limited literacy (0–1)	Overall NVS-PK score	196 (49.0)	44.0–54.0
Category 2: possibility of limited literacy (2–3)	Overall NVS-PK score	128 (32.0)	27.5–36.8
Category 3: adequate literacy (4–6)	Overall NVS-PK score	76 (19.0)	15.3–23.2
Combined limited or marginal (0–3)	Overall NVS-PK score	324 (81.0)	76.8–84.7
Item-level accuracy (Items B1–B6)			
B1: energy in the whole pack (148 × 5 = 740 kcal)	Multi-step multiplication	103 (25.8)	21.5–30.3
B2: servings within a 38 g carbohydrate allowance	Division	117 (29.2)	24.8–34.0
B3: change in daily total fat intake	Subtraction/addition	89 (22.2)	18.3–26.6
B4: sodium as % of daily limit (140 ÷ 2000 = 7%)	Percentage calculation	59 (14.8)	11.4–18.6
B5: safety for a wheat (gluten) allergy	Document/allergen literacy	267 (66.8)	61.9–71.4
B6: naming the wheat/gluten ingredient	Document/allergen literacy	120 (30.0)	25.5–34.8

*N =* 400. NVS-PK, culturally adapted Newest Vital Sign—Pakistan; scores range 0–6. Confidence intervals for proportions are exact (Clopper–Pearson) intervals [39]. Mean total score 1.89 (SD = 1.71); applied-numeracy subscore (B1–B4) 0.92 (SD = 1.16) of 4; document-literacy subscore (B5–B6) 0.97 (SD = 0.80) of 2. Internal consistency: Cronbach’s α = 0.733 overall, 0.637 (numeracy subscale), 0.632 (document-literacy subscale). Item difficulty was not equal across the six items, Cochran’s Q(5) = 406.96, *p* < 0.001.

**Table 3:** Multiple linear regression on NVS-PK total score (*N =* 400).

Predictor	Model 1: B (95% CI)	Model 2: B (95% CI)	β	*p*
Age (per year)	−0.013 (−0.031, 0.005)	−0.014 (−0.032, 0.004)	−0.067	0.122
Male (ref. female)	−0.007 (−0.298, 0.284)	0.002 (−0.287, 0.291)	0.001	0.989
Bachelor’s degree (ref. Intermediate or below)	1.157 (0.791, 1.524)	1.062 (0.692, 1.432)	0.278	**<0.001**
Postgraduate (ref. Intermediate or below)	1.797 (1.341, 2.252)	1.638 (1.173, 2.102)	0.343	**<0.001**
PKR 50,000–74,999 (ref. below PKR 50,000)	0.126 (−0.272, 0.524)	0.075 (−0.323, 0.474)	0.019	0.710
PKR 75,000 or above (ref. below PKR 50,000)	0.586 (0.205, 0.966)	0.412 (0.014, 0.810)	0.120	**0.043**
Tier 2 (ref. Tier 1)	—	−0.358 (−0.720, 0.005)	−0.099	0.053
Tier 3 (ref. Tier 1)	—	−0.591 (−0.995, −0.186)	−0.163	**0.004**
Adjusted R^2^	—	0.259	—	0.271
R^2^ change	—	—	—	0.015 (*p* = 0.015)

Dependent variable: NVS-PK total score (0–6). Model 1 contains age, gender, educational attainment and household income; Model 2 adds retail tier. Reference categories: female (gender); Intermediate education or below (education); below PKR 50,000 (household income); Tier 1 (retail tier). β and *p* are reported for Model 2. Durbin–Watson = 1.932; variance inflation factors < 2 throughout. Bold *p*-values indicate *p* < 0.05.

**Table 4:** Binary logistic regression on adequate nutrition label literacy (*N =* 400).

Predictor	Adjusted OR (95% CI)	*p*
Age (per year)	1.005 (0.971, 1.040)	0.779
Male (ref. female)	0.877 (0.503, 1.529)	0.644
Bachelor’s degree (ref. Intermediate or below)	4.23 (2.04, 8.79)	**<0.001**
Postgraduate (ref. Intermediate or below)	8.72 (3.88, 19.60)	**<0.001**
PKR 50,000–74,999 (ref. below PKR 50,000)	0.91 (0.36, 2.32)	0.849
PKR 75,000 or above (ref. below PKR 50,000)	1.39 (0.60, 3.23)	0.447
Tier 2 (ref. Tier 1)	0.43 (0.22, 0.84)	**0.013**
Tier 3 (ref. Tier 1)	0.58 (0.26, 1.32)	0.196

Dependent variable: adequate nutrition label literacy (NVS-PK score 4–6) versus non-adequate (0–3). Single model containing the complete predictor set (same reference categories as Table 3). Hosmer–Lemeshow χ^2^(8) = 7.48, *p* = 0.486; Nagelkerke R^2^ = 0.253; 82.0% of cases correctly classified. Bold *p*-values indicate *p* < 0.05.

**Table 5:** Ranked prevalence of perceived barriers to label use, by literacy category (*N =* 400).

Rank	Perceived Barrier	n (%)	Differs by Literacy Category?
1	Technical or scientific terminology confusing	281 (70.2)	No (*p* = 0.386)
2	Lack of time/hurry while shopping	256 (64.0)	No (*p* = 0.595)
3	Calculations and percentage conversions too complex	228 (57.0)	Yes (*p* < 0.001)
4	Information overload/cluttered layout	179 (44.8)	No (*p* = 0.679)
5	Font size too small	161 (40.2)	No (*p* = 0.886)
6	Brand loyalty/trust in familiar manufacturer	161 (40.2)	No (*p* = 0.739)
7	Price and expiry override nutrition	157 (39.2)	Yes (*p* = 0.004)
8	English-only labelling (no Urdu)	148 (37.0)	Yes (*p* < 0.001)
9	Low perceived utility of reading labels	129 (32.2)	No (*p* = 0.147)
10	Difficulty locating the nutrition panel	107 (26.8)	No (*p* = 0.193)

Multi-select inventory; participants could endorse any number of items, and percentages are calculated on the full sample of *N =* 400. Mean barriers endorsed per participant: 4.52 (SD = 1.51). Cochran’s Q across the ten items = 284.73, df = 9, *p* < 0.001, confirming that items are not endorsed at equal rates. “Differs by literacy category” reports the chi-square test (df = 2) of endorsement across the three NVS-PK literacy categories. An eleventh free-text “other” category, endorsed by 5.5% (*n =* 22), is excluded from ranking and from Cochran’s Q.

## Data Availability

The data presented in this study are available on request from the corresponding author, due to institutional policy of the first author.
